# The majority of β-catenin mutations in colorectal cancer is homozygous

**DOI:** 10.1186/s12885-020-07537-2

**Published:** 2020-10-28

**Authors:** Alexander Arnold, Moritz Tronser, Christine Sers, Ayel Ahadova, Volker Endris, Soulafa Mamlouk, David Horst, Markus Möbs, Philip Bischoff, Michael Kloor, Hendrik Bläker

**Affiliations:** 1 Institute of Pathology, Charité - Universitätsmedizin Berlin, Virchoweg 15 / Charitéplatz 1, 10117 Berlin, Germany; 2 Department of Applied Tumor Biology, Institute of Pathology, University of Heidelberg; Clinical Cooperation Unit Applied Tumor Biology, German Cancer Research Center (DKFZ); Molecular Medicine Partnership Unit (MMPU), University Hospital Heidelberg, Heidelberg, Germany; 3 Department of General Pathology, Institute of Pathology, University Hospital Heidelberg, Heidelberg, Germany; 4 Present address: Institute of Pathology, Universitätsklinikum Leipzig, Leipzig, Germany

**Keywords:** Colorectal cancer (CRC), ß-catenin (*CTNNB1*), E-cadherin

## Abstract

**Background:**

β-catenin activation plays a crucial role for tumourigenesis in the large intestine but except for Lynch syndrome (LS) associated cancers stabilizing mutations of *β-catenin* gene (*CTNNB1)* are rare in colorectal cancer (CRC). Previous animal studies provide an explanation for this observation. They showed that *CTNNB1* mutations induced transformation in the colon only when *CTNNB1* was homozygously mutated or when membranous β-catenin binding was hampered by E-cadherin haploinsufficiency. We were interested, if these mechanisms are also found in human *CTNNB1* mutated CRCs.

**Results:**

Among 869 CRCs stabilizing *CTNNB1* mutations were found in 27 cases. Homo- or hemizygous *CTNNB1* mutations were detected in 74% of *CTNNB1* mutated CRCs (13 microsatellite instabile (MSI-H), 7 microsatellite stabile (MSS)) but only in 3% (1/33) of extracolonic *CTNNB1* mutated cancers. In contrast to MSS CRC, *CTNNB1* mutations at codon 41 or 45 were highly selected in MSI-H CRC. Of the examined three CRC cell lines, β-catenin and E-cadherin expression was similar in cell lines without or with hetereozygous *CTNNB1* mutations (DLD1 and HCT116), while a reduced E-cadherin expression combined with cytoplasmic accumulation of β-catenin was found in a cell line with homozygous *CTNNB1* mutation (LS180). Reduced expression of E-cadherin in human MSI-H CRC tissue was identified in 60% of investigated cancers, but no association with the *CTNNB1* mutational status was found.

**Conclusions:**

In conclusion, this study shows that in contrast to extracolonic cancers stabilizing *CTNNB1* mutations in CRC are commonly homo- or hemizygous indicating a higher threshold of β-catenin stabilization to be required for transformation in the colon as compared to extracolonic sites. Moreover, we found different mutational hotspots in *CTNNB1* for MSI-H and MSS CRCs suggesting a selection of different effects on β-catenin stabilization according to the molecular pathway of tumourigenesis. Reduced E-cadherin expression in CRC may further contribute to higher levels of transcriptionally active β-catenin, but it is not directly linked to the *CTNNB1* mutational status.

**Supplementary information:**

**Supplementary information** accompanies this paper at 10.1186/s12885-020-07537-2.

## Background

Molecular biological studies over the last 4 decades have unraveled the multidimensional role of β-catenin (encoded by the *CTNNB1* gene) in embryological development and carcinogenesis [1]. Physiologically, β-catenin acts as a regulator of gene expression in the WNT-signaling pathway and as a crucial component of cell-cell-adhesion. In short, activating WNT signaling results in cytoplasmatic accumulation of free β-catenin and its subsequent nuclear translocation, where it functions together with other coactivators as an active transcriptional complex for the expression of WNT target genes [2, 3]. On the other hand, β-catenin forms a complex with E-cadherin, which mediates cell-cell-adhesion, thereby maintaining epithelial tissue integrity [4, 5].

Frequent heterozygous mutations in hotspot region of exon 3 in *CTNNB1* have been described for different tumour entities such as hepatocellular carcinoma [6], solid pseudopapillary tumour [7] and desmoid fibromatosis [8]. In contrast, *CTNNB1* mutations are rare in CRC. Here, the activation of the WNT pathway appears to be mostly driven by a bi-allelic alteration of the *APC* gene resulting in decreased β-catenin degradation [9]. However, alterations in *CTNNB1* itself represent a well characterized alternative to the canonical *APC* driven pathway activation [10]. Deletions and mutations in the hotspot of exon 3 lead to a loss of serine/threonine phosphorylation sites impeding the degradation of ß-catenin by the destruction complex, thereby increasing cytoplasmic β-catenin protein levels [11]. Moreover, in CRCs, *APC* and *CTNNB1* mutations are almost always mutually exclusive and associated with different genetic signatures [12]. While *APC* mutations are found more frequently in microsatellite stable (MSS) CRCs, *CTNNB1* mutations are commonly associated with microsatellite instability (MSI-H) particularly in cancers from Lynch syndrome patients [13, 14].

Besides the activation of the WNT pathway, alterations of *CTNNB1* may influence E-cadherin-mediated cell-cell-adhesion. These aberrations are known to be associated with reduced protein levels of membranous E-cadherin and the expression of markers of epithelial-mesenchymal transition (EMT), thereby possibly facilitating metastatic spread of malignant cells [15, 16]. Moreover, Huels et al. (17) have linked these complex interactions between ß-catenin and E-cadherin to the different tumourigenic potential of *CTNNB1* mutations in the colon compared to other anatomic locations. In their animal model study they show that heterozygous *CTNNB1* mutations are drivers of tumourigenesis in the small intestine while in the large bowel homozygous mutations of *CTNNB1* are necessary for tumour development.

In order to study the characteristics of *CTNNB1* mutations in CRC and their association with altered E-cadherin expression, we evaluated 869 CRCs for *CTNNB1* mutations and analysed the expression of E-cadherin and β-catenin in three cell lines with *CTNNB1* wildtype (DLD1), heterozygous (HCT 116), and homozygous mutations (LS 180).

## Methods

### CRC cohorts

#### Two CRC cohorts were analysed

Cohort 1 consisted of 839 CRCs panel sequenced for diagnostic purposes from January 2015 to December 2017 in the Department of Pathology, Charité University Medicine Berlin. Samples were designated for companion (a theranostic testing of the K-RAS/N-RAS status that indicate a patient’s response to an EGFR-inhibitor) diagnostics (826 CRC) or for *BRAF* analysis to distinguish sporadic *BRAF* mutated from potentially Lynch Syndrome associated MLH1 deficient MSI-H CRCs without *BRAF* mutation (13 MLH1 deficient MSI-H CRCs). MSI status was known for 221 of the CRCs. Thirty one CRCs were MSI-H and 190 CRCs were MSS.

Cohort 2 included CRCs from a scientific study on somatic mutations in 30 CRCs associated with Lynch syndrome (LS). LS is caused by germline mutations in one of the DNA mismatch repair genes MLH1, MSH2, MSH6, or PMS2. In the present cohort LS associated CRCs were deficient for the DNA mismatch repair proteins MLH1, MSH2, MSH6, or PMS2 in 13, 11, 4, and 2 LS CRCs, respectively.

For 21 cases of cohort 2 data on the presence or absence of *CTNNB1* mutations has been reported previously [17].

The study was approved by the ethics committees of Berlin and Heidelberg (Application Berlin EA4/003/19 and EA1/413/16 and Heidelberg S-583/2016).

### Extracolonic cancer cohort

To compare the findings of homozygous *CTNNB1* in CRCs with those in extracolonic cancers we analysed panel sequenced lung cancers and melanomas. The Lung cancer cohort consisted of 23 non-small cell lung cancer analysed for *EGFR* mutations and the melanoma cohort of 10 metastasized melanomas analysed for *BRAF* mutations. Cancers were panel sequenced for therapy predictive purposes from January 2015 to December 2017 in the Department of Pathology, Charité University Medicine Berlin.

### Scoring of mono- and biallelic CTNNB1 mutations in CRC

Biallelic mutations are defined by mutations in both, the maternal and paternal allele copy. Both copies may harbour the same mutation (homozygous), one copy may be mutated while the second copy is lost (hemizygous), or both copies may be affected by different mutations (compound heterozygous).

While detection of compound heterozygous mutations is straight forward, the interpretation of homo- or hemizyous mutations in cancer tissue is hampered by admixed non-neoplastic cells, which dilute tumorous DNA and thus lower the proportion of cancerous mutations. In routine practice, DNA isolated from cancers is “contaminated” by 20–70% of DNA from non-neoplastic cells. To score *CTNNB1* mutations for mono- or biallelic mutations we followed expected mutational allele frequencies (AF) for tumor specific somatic mutations in samples with varying tumor cell purity as described previously [18].

In brief, we calculated the expected AFs of heterozygous and homo- or hemizygous mutations for different gene copy numbers and tumor cell purities. We defined mutations in genes typically mutated heterozygously in CRC as heterozygous reference mutations. For this purpose mutated AFs of oncogenes (*KRAS, PIK3CA*) where used as reference mutations for comparison with *CTNNB1*. In cases without mutations in oncogenes, the AF of mutated *FBXW7* was evaluated. Despite being considered a tumor suppressor, *FBXW7* mutations are almost always heterozygous in CRC [19].

We reasoned that irrespective of the tumor cell purity the AF of a homozygous mutation is twice as high as the AF of a heterozygous mutation. Accordingly, a *CTNNB1* mutation was scored as biallelic/homozygous when its AF was twice as high as the AF of a reference gene (criterion for homozygosity). Heterozygous mutations were scored when the AF of the *CTNNB1* was as high as that of the reference mutation.

In *CTNNB1* mutated CRCs without reference mutations, we concluded that AFs ≥50% cannot result from heterozygous mutations. In these cases we considered homo- or hemizygous mutations. Biallelic mutations with retention of a wild type copy ^(mut/mut/wildtype)^ were considered unlikely due to the demonstration of wild type loss by RNA sequencing, the high tumour cell purity required to exceed an AF of 50%.

We therefore scored *CTNNB1* mutations in CRCs without reference mutations as biallelic/homo- or hemizygous when the AF was ≥50% (criterion for homo-or hemizygous mutation) and as heterozygous when the AF of the *CTNNB1* mutation was < 50%.

### Statistical analysis

Statistical analysis was performed using RStudio version 1.1.463 based on the statistical language R version 3.5.1. The chi square test was used to calculate significance for categorical variables. The level of significance was set at *p* < 0.05.

### Next generation sequencing

After microscopic identification of areas with highest tumor cell concentration, DNA was isolated using a commercial DNA Extraction Kit (DNeasy; Qiagen) following the manufacturer’s protocol.

In cohort 1, multiplex PCR-based amplicon high-throughput sequencing was performed using the Ion Torrent system (ThermoFisher Scientific) according to the manufacturer’s recommendations. Briefly, 10 ng of genomic DNA was used for library construction with the Ion AmpliSeq Colon and Lung Cancer Panel v2 and the Ion AmpliSeq Library Kit 2.0 (ThermoFisher). Library quantification was carried out on the StepOne Plus Real-Time PCR System employing the Ion Library TaqMan Quantitation Kit (both ThermoFisher). Emulsion PCR and subsequent enrichment was done on the Ion Chef instrument (ThermoFisher). Data analysis was performed using the Sequence Pilot software (JSI medical systems GmbH). Mean sequencing depth for *CTNNB1* exon 3 and commonly heterozygous CRC mutations (*KRAS, PIK3CA, FBXW7*) was 1816 (range 569–4241) and 1308 (range 482–3671), respectively.

CRCs from cohort 2 were analysed with more extended customized panels as previously reported [17]. Mean sequencing depth for *CTNNB1* exon 3 and commonly heterozygous CRC mutations (*KRAS, PIK3CA, FBXW7*) was 6144 (range 1891–11,334) and 3473 (range 1342–9467), respectively.

### RNA sequencing of CTNNB1

To confirm the homozygosity of *CTNNB1* mutations, RNA was isolated from slides obtained from tumor tissue blocks using the RNeasy FFPE-Kit (Qiagen, Germany) according to the manufacturer’s protocol.

Primers spanning from exon 1 to exon 4 of the *CTNNB1* mRNA were used.

RNA *CTNNB1* forward 5′-GTC GAG GAC GGT CGG ACT-3′.

RNA *CTNNB1* reverse 5′-CAG GAC TTG GGA GGT ATC CA-3′.

For single step generation of cDNA and PCR amplification we used the Invitrogen one step RT PCR KIT with platinum Taq (Fisher Scientific) according to the manufacturer’s protocol. PCR consisted of a single reverse transcription step at 55 °C for 30 min, followed by denaturation at 94 °C for 2 min and 40 cycles each consisting of a denaturation (94 °C for 15 s), annealing (60 °C for 30 s), and an extension step (68 °C for 1 min). PCR products were analysed by Sanger sequencing.

### Cell culture

Colorectal cancer cell lines DLD1 were purchased from Horizon, HCT116 and LS180 from ATCC. The reported mutational status of *CTNNB1* (wt/wt for DLD1, del45S/wt for HCT116, and S45F/S45F for LS180) (https://www.ncbi.nlm.nih.gov/pubmed/24755471) was confirmed by DNA Sanger sequencing. DLD1, HCT116, and LS180 cells were cultured in RPMI medium, DMEM supplemented with 2 mM Ultraglutamine (Lonza), or EMEM, respectively, each containing 10% FCS and 100 U/ml penicillin-streptomycin (Biochrom).

### Immunostaining of cell cultures

After reaching 70–80% confluence, cells were harvested for immunofluorescence staining. Therefore, cells were washed with PBS twice and fixated with 4% formaldehyde for 30 min. Subsequently, cells were scratched from the plate using a spatula, and transferred in PBS into 15 ml Falcon tubes. Scratched cells in PBS were centrifuged for 2 min at 1200 rpm. The pellet was resuspended in histogel (ThermoScientific) at 70 C°, transferred into a histo cassette, embedded into paraffin, and cut in 3–4 μm sections. For deparaffinization of paraffin sections, slides were incubated in xylene for 5 min three times followed by rehydration in descending dilutions of ethanol in water (100, 90, 80, 70, 0%) for 5 min each at room temperature. For antigen unmasking, slides were incubated in citrate antigen retrieval buffer (82 mM trisodium citrate dihydrate, 18 mM citric acid monohydrate, adjust to pH 6.0) for 20 min at 100 °C, and unspecific binding sites were blocked by incubation with Peroxidase-Blocking Solution (Dako) for 30 min at room temperature. Slides were incubated with primary antibodies (Rabbit anti-β-catenin (#9562 Cell Signaling) 1:400, Mouse anti-E-cadherin (#14472 Cell Signaling) 1:200) overnight at 4 °C. For immunostaining, slides were subsequently incubated with secondary antibodies (Donkey anti-rabbit IgG Alexa Fluor 488, Goat anti-mouse IgG Alexa Fluor 555 (both Invitrogen), both 1:600) for 1 h at room temperature. Cell nuclei were visualized by incubation with 0.1 μg/ml DAPI in TBS. Slides were covered with Vectashield® mounting medium and coverslips. Images were acquired using an Axiovert flouresence microscope equipped with an Axiocam 506 color camera (both Zeiss). All incubation steps were followed by washing the slides with TBS three times. Antibodies were diluted in Antibody Diluent (Zytomed).

### Western blot analysis of cell lines

Protein was retrieved at 70–80% confluent growth. After washing twice with PBS, cells were scratched from the plates, transferred into a 1.5 ml tube, and centrifuged for 5 min at 5000 rpm at 4 °C. The pellet was resuspended in M-Per buffer (Thermo Scientific) supplemented with cOmplete protease inhibitor mix (Roche) and Phosphostopp phosphatase inhibitor mix (Roche). After sonification for 5 min and centrifugation at 13000 rpm for 10 min at 4 °C, protein concentration in the supernatant was measured using the BCA Protein Assay Kit (Pierce) according to the manufacturer’s protocol. Twenty microgram of protein in 6x loading buffer (300 mM Tris, 600 mM DDT, 12% SDS, 60% glycerol, bromphenol blue according to desired intensity) was heated to 95 °C for 10 min, cooled on ice and loaded on 2% SDS stacking/10% SDS separation gels. PageRuler Prestained Protein Ladder (Thermo Scientific) was used as protein weight standard.

Electrophoresis was run at 100 V. Subsequently, proteins were transferred to nitrocellulose membranes by semi-dry blotting between filter papers soaked with blotting buffer (25 mM Tris, 150 mM glycine, 10% methanol). Nitrocellulose membranes were blocked with 5% milk powder in TBST for 30 min. Incubation with primary antibodies (Rabbit anti-β-catenin (#9562 Cell Signaling), Mouse anti-E-cadherin (#14472 Cell Signaling), Rabbit anti-β-tubulin (#2146 Cell Signaling), all 1:1000) was carried out overnight at 4 °C, incubation with secondary antibodies (horse anti-mouse HRP-linked IgG; 7076S or goat anti-rabbit HRP-lined IgG 7074S, both Cell Signaling), was carried out for 1 h at room temperature. Protein bands were detected using the ECL Western Blot Detection Reagent (Amersham) according to the manufacturer’s protocol. All incubation steps were followed by washing the nitrocellulose membranes with TBST three times. Antibodies were diluted in 5% milk powder in TBST.

### Immunohistochemistry of human CRC samples

Formalin-fixed and paraffin-embedded tissue samples of CRC were cut into 4 μm sections. A BenchMark XT immunostainer (Ventana Medical Systems, Tucson, AZ) was used for subsequent immunohistochemical staining. For antigen retrieval, sections were incubated in CC1 mild buffer (Ventana Medical Systems, Tucson, AZ) for 30 min at 100 °C. Sections were stained with anti-E-cadherin antibody (clone 4A2C7, 1:50, Zytomed) for 60 min at room temperature, and visualized using the avidin-biotin complex method and DAB. Cell nuclei were counterstained by incubation with hematoxylin and bluing reagent (Ventana Medical Systems, Tucson, AZ) for 12 min each.

Tissue blocks for immunohistochemistry were available for 12 *CTNNB1* mutated CRC and 5 CRC with *CTNNB1* wild type. All 17 CRCs were MSI-H.

The stains were evaluated using an Olympus BC50 microscope and Olympus PLN 4X/0.1 Plan Achromat Objectives (Olympus Europe Holding GmbH, Hamburg, Germany). Histological images were acquired with the digital camera moticam 3.0 mp (Motic Instruments Inc., California, USA) and Motic Images Plus software (Motic instruments Inc.).

## Results

### CTNNB1 mutations in CRC

Among 839 CRCs from the diagnostic cohort we detected 16 cases with *CTNNB1* mutations (1.9%), which comprised 7 MSI-H and 9 MSS tumors. The 7 *CTNNB1* mutated MSI-H CRCs included 3 tumors analysed for LS diagnostics and one metastasized tumor from an LS patient analysed for companion diagnostics. LS status was unknown in the remaining 3 *CTNNB1* mutated MSI-H CRC sequenced for therapy prediction. None of the *CTNNB1* mutated MSI-H CRCs carried a *BRAF* mutation.

Among 30 LS CRCs from the scientific cohort we detected 11 *CTNNB1* mutations (37%). Nine CRCs were MLH1 deficient and one tumor each was MSH2 and PMS2 deficient.

Among the total of 27 *CTNNB1* mutated CRCs of both cohorts, *CTNNB1* mutations in 13 CRCs (2 MSS, 11 MSI-H) were scored as biallelic/homozygous. Values for *CTNNB1* AFs relative to the AFs of reference mutations were highly concordant with expected values and ranged between 1.9 and 2.1. Only *CTNNB1* mutations in two CRCs displayed relative values of 2.4 and 3.1. Given a near perfect match of the relative AFs with that calculated for 3 and 4 mutated allele copies in the absence of wild type alleles, respectively, these tumours were also scored as homozygous.

In 10 out of 13 CRCs with homozygous *CTNNB1* mutations the AFs of mutated *CTNNB1* exceeded 50%.

*CTNNB1* mutations in 7 CRCs without a reference mutation had AFs > 60% (range 62–78%) and were scored as biallelic/ homo- or hemizygous.

*CTNNB1* mutations in 7 CRCs were scored monoallelic/heterozygous.

The results are summarized in Table 1 and the supplementary Table 1.

**Table 1:** Summarized results of *CTNNB1* mutations both, diagnostic and scientific cohorts. *CTNNB1* mutated CRCs of diagnostic cohort were further split according to MSI status. For detailed description of individual cases, see Supplementary Table 1

Cohort	Diagnostic	Diagn. MSS	Diagn. MSI-H	Scientific
*CTNNB1* mutated	16/839 (1.9%)	9	7	11/30 (37%)
biallelic	12/16	7/9	5/7	8/11
homozygous	6	2	4	7
homo- or hemizygous	6	5	1	1
heterozygous	4	2	2	3

### CTNNB1 RNA sequencing

**Fig. 1:**
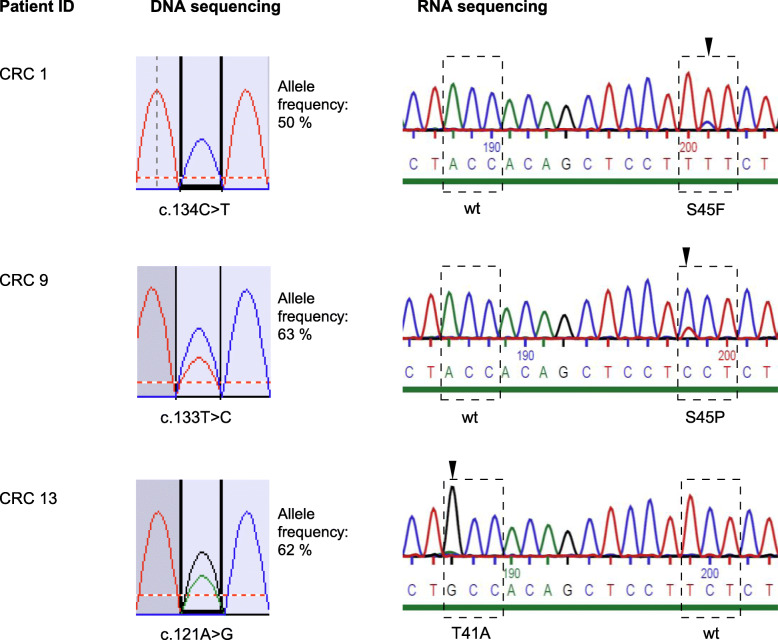
Comparison of mutated *CTNNB1* allele frequencies as detected by DNA sequencing and RNA sequencing in 3 MSI-H cancers (CRC1, 9, 13). Since β-catenin is strongly expressed in cancer cells but not in contaminating lymphocytes, the level of nucleic acid contamination by lymphocytes is less for RNA than for DNA. Faint residual wild type alleles seen in CRC1 and CRC9 were considered to result from β-catenin expressing blood vessels

The homozygous nature of *CTNNB1* mutations was exemplarily confirmed by RNA sequencing of *CTNNB1* in four cases, in which DNA sequencing revealed *CTNNB1* mutations with AFs ranging from 50 to 63% (Fig. 1). In all four cases, wild type *CTNNB1* RNA was absent or minimally present. Remnants of wild type *CTNNB1* were considered to result from expression of *CTNNB1* by stromal cells.

### Zygosity of CTNNB1 mutations in the extracolonic cancer cohorts

None of the 23 lung cancers with *CTNNB1* mutations fulfilled the criterion for biallelic/ homozygous mutation and one tumor displayed a *CTNNB1* AF of 57% and thus was scored as biallelic/homo- or hemizygous. The remaining 22 lung cancers and all 10 *CTNNB1* mutated melanomas were scored as monoallelic/heterozygous.

Using the chi square test we calculated the significance of the frequency of homo/hemizygous mutations in CRC vs. lung/melanoma cancers resulting in a *p*-value < 0,001.

### Comparison of CTNNB1 mutational hotspots in MSI-H and MSS cancers

In accordance with previous reports, we found the frequency of *CTNNB1* mutations to be higher in MSI-H CRC than in MSS CRC and our data indicate that among the MSI-H CRC *CTNNB1* mutations are mainly contributed by LS-associated tumors. We also discovered that not only the frequency, but also the type of *CTNNB1* mutations differed between MSI-H and MSS CRCs. All MSI-H cancers displayed *CTNNB1* mutations affecting either residue T41 or S45. The majority of alterations were missense mutations while two cases displayed 3 bp deletions at codon 45 (S45del) and one tumour harbored a 12 bp deletion (delS45-G48).

**Fig. 2:**
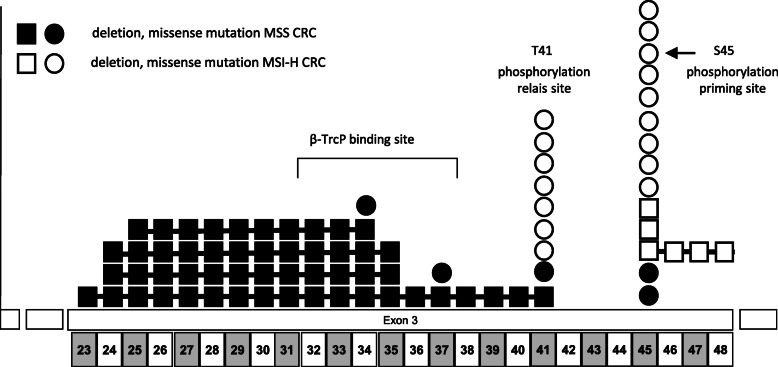
Differences between *CTNNB1* mutations in MSI-H and MSS cancers. In MSI-H tumours *CTNNB1* mutations cluster exclusively at codons 41 and 45 and mainly consist of missense mutations. In MSS cancers *CTNNB1* mutations frequently cover the ß-TrcP binding site and consist of larger in frame deletions. Captation: squares white: deletion, MSI-H; squares black: deletion, MSS; circles white: missense mutation, MSI-H; circles black: missense mutation, MSS

In MSS cancers, mutations were more evenly distributed among mutational hotspots. We detected in-frame deletions in 4 cases overlapping at codons 25–37, and 5 missense mutations at codon 34, 37, 41 (2), and 45 (Table 1, Fig. 2).

Using the chi square test we calculated significance of the hotspot mutations in MSI vs. MSS CRCs resulting in a *p*-value < 0,030.

### β-Catenin and E-cadherin expression in cell lines

**Fig. 3:**
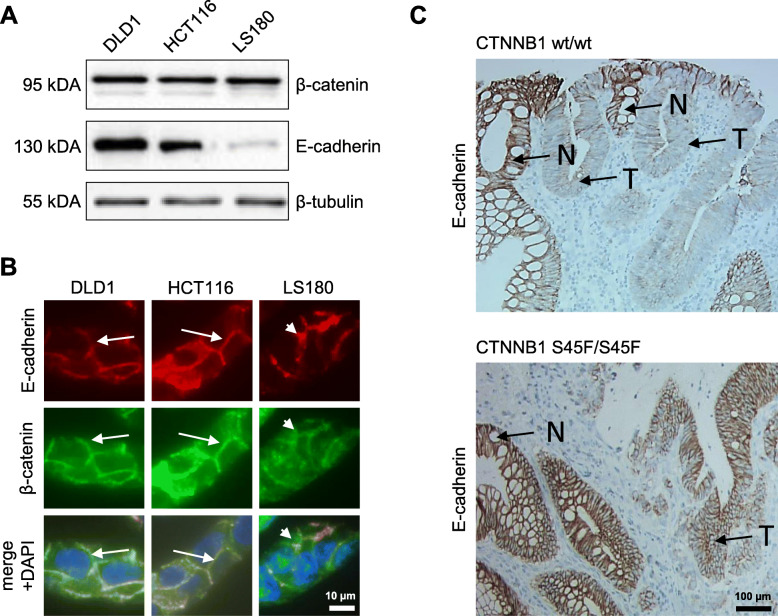
E-cadherin and β-catenin expression in colorectal cancer cell lines DLD1 (CTNNB1 wt/wt), HCT116 (CTNNB1 S45del/wt), and LS180 (CTNNB1 S45F/S45F). **a** Strongly reduced E-cadherin expression in LS180 as compared to DLD1 and HCT116. **b** E-cadherin and β-catenin immunoflourescence staining showing reduced levels and aberrant membranous localization of E-cadherin only for LS180 cells Distinct membranous colocalization of E-cadherin and β-catenin in DLD1 and HCT116 (long white arrows) and dot-like discontinuous expression of E-cadherin and β-catenin in LS180 (short arrows), (original magnification ×400). **c** Immunohistochemistry of E-cadherin in tumour samples from CRC patients. Upper image. Tumour specific (T) reduction of E-cadherin expression in comparison to non neoplastic crypts (N) in a *CTNNB1* wildtype CRC. Lower image: Normal tumor specific E-cadherin expression in a *CTNNB1* mutated CRC (original magnification × 200)

In order to gain an insight into the association of *CTNNB1* mutations with E-cadherin expression, we studied β-catenin and E-cadherin levels in different MSI-H CRC cell lines by western blot analysis and immunofluorescence microscopy. Interestingly, we observed that β -catenin protein levels were similar in all three cell lines analyzed (DLD1: wildtype/wildtype, HCT116: wildtype/S45del, LS180: S45F/S45F). However, E-cadherin protein levels were significantly lower in LS180 than in DLD1 and HCT116 cells. This difference in E-cadherin protein levels could also be seen by immunohistochemistry. We further observed that LS180 cells displayed an incomplete, somewhat patchy E-cadherin distribution along the cell membrane while DLD1 and HCT116 cells showed a complete and distinct membrane staining of E-cadherin. Immunofluorescence with co-staining of β-catenin and E-cadherin confirmed the irregular distribution pattern E-cadherin in LS180 cells seen by immunohistochemistry. It further showed that this phenotype is associated with a diffuse cytoplasmic localization of β-catenin. In contrast, in DLD1 and HCT116 cells, β-catenin and E-cadherin are mainly co-localized along the cell membrane (Fig. 3) while only single cells in HCT116 displayed cytoplasmic and nuclear staining of β-catenin.

### E-cadherin expression in human CRCs with and without CTNNB1 mutation

In vitro, we found that homozygous *CTNNB1* mutation at residue S45 might perturb E-cadherin-mediated cell-cell adhesion. Hence, we questioned if *CTNNB1* mutations found in CRCs also have an impact on E-cadherin expression.

Therefore, we analyzed the E-cadherin expression in 17 MSI-H CRCs with known *CTNNB1* mutational status (10 homo- or hemizygous, 2 heterozygous, 5 wildtype) Strong reduction of E- Cadherin was found in 11 cancers while 6 CRCs displayed an expression similar to that in surrounding epithelium (Fig. 3). Aberrant expression of E-cadherin, however, was neither exclusive to *CTNNB1*-mutated tumours nor did all *CTNNB1* mutated CRCs display reduced E-cadherin expression. Three out of 5 CRCs (60%) without *CTNNB1* mutations and 8 of 12 CRCs (66%) with *CTNNB1* mutations showed a reduced concentration of membranous E-cadherin while normal E-cadherin expression was found in 2 CRCs without and 4 CRCs with *CTNNB1* mutations.

## Discussion

This is the first study systematically analysing CRCs for biallelic mutations in the *β-catenin* gene. Using absolute and relative allele frequencies of *CTNNB1* mutations to score zygosity we detected 71% of *CTNNB1* mutated CRCs (20/28) harbouring biallelic mutations mainly due to mutational homozygosity.

One limitation of a computational method in scoring zygosity is that not all factors influencing mutated allele frequency are considered. For example copy number gains might have affected the mutated *CTNNB1* allele and thus elevated the mutated allele frequency. Moreover, we cannot exclude that tumour heterogeneity lowered the mutated allele frequencies of the reference mutations, particularly in *PIK3CA*, and thus could have resulted in a false interpretation of *CTNNB1* mutational homozygosity.

To exclude a significant impact of these limitations we therefore compared the results in CRC to those in extracolonic tumour types, which should be affected equally by a systematic methodical limitation. In these extracolonic cancers, however, the computational method did not reveal homozygous *CTNNB1* mutations. Among 33 extracolonic *CTNNB1* mutated cancers we detected only a single lung cancer fulfilling the criterion for a hemizygous mutation (4%). These data clearly delineate the *CTNNB1* mutational zygosity findings in CRCs from those in cancers outside the colon (74% versus 4%; *p* < 0,001).

While *CTNNB1* alterations have been investigated extensively in human cancers, only a few studies have drawn attention to biallelic mutations. Reports of homozygous *CTNNB1* mutations are found for parathyroid adenoma [20] and for two colorectal adenomas [21, 22]. Moreover, Rebouissou et al. recently reported homo- and hemizygous mutations of *CTNNB1* in hepatocellular carcinoma. In that study, double hit mutations were restricted to codon 45 while mutations at β-TRCP binding site (codons 32–37) and at codon 41 were heterozygous [23]. Using functional assays, the authors found that mutations at codon 45 only led to a weak activation of β-catenin compared to mutations at codon 41 and at the β-TRCP binding site, which resulted in moderate and strong activation, respectively. It was concluded that homozygous mutations at S45 are necessary to confer an oncogenic effect strong enough to induce a malignant tumour.

A similar explanation may also apply to *CTNNB1-*mutated CRC. In previous animal studies, Huels et al. found that a single *CTNNB1* mutation drives tumourigenesis in the small bowel, but is insufficient to induce transformation in the colon [24]. The discrepant effect in the small and the large bowel was explained by a higher expression of E-cadherin which binds excessive β-catenin to the membrane and thus compensates for the oncogenic effect of single hit *CTNNB1* mutations in the colon. The tumour suppressive effect of E-cadherin, however, is overcome in the presence of homozygous *CTNNB1* mutations indicating that threshold exceedance of mutated β-catenin levels also plays a role in colorectal neoplasia.

Apart from doubling the number of mutated alleles in tumour cells, homozygous *CTNNB1* mutations in colorectal cancer also result in a loss of wild type β-catenin, a finding usually associated with inactivation of a tumour suppressor. The presence of *CTNNB1* mutational homozygosity in cancers at a specific site, like the colon, may suggest that oncogenic activation acts in concert with a loss of tissue-dependent tumour suppression. One such β-catenin function results from its engagement in the formation of adherens junctions by mediating the mechanical link of E-cadherin to the actin cytoskeleton. Two previous analyses on HCT116, a cell line which carries a heterozygous *CTNNB1* mutation at codon 45, reported that knockdown of the wild type copy resulted in the loss of E-cadherin-mediated adhesion function [25, 26]. In accordance with these findings, we detected reduced E-cadherin levels and aberrant membranous distribution exclusively in LS180 cells, the only CRC cell line we investigated, which harbours a homozygous *CTNNB1* mutation. Further E-cadherin analysis in human cancers, however, drew a more complex picture. Reduced E-cadherin expression was neither exclusive to CRCs with homozygous *CTNNB1* mutations, nor did all CRCs with homozygous mutations exhibit an aberrant E-cadherin expression. Thus, our data do not show an exclusive link between *CTNNB1* mutations and reduced E-cadherin expression in human CRC tissue.

The reason for the divergent results in cell culture and human tissue analyses is not clear. It is possible, that the reduction of E-cadherin expression promotes tumour development by enhancing β-catenin nuclear activity, yet is caused by mechanisms different from *CTNNB1* homozygous mutations in both, CRC cell line LS180 and CRC tissue. Beside transcriptional downregulation of E-cadherin by a factor acting in concert with β-catenin stabilization one such mechanism could be the mutational inactivation of δ-catenin (p120), a protein that stabilizes E-Cadherin at the cell membrane. Interestingly, biallelic mutations of the δ-catenin gene, *CTNND1*, have recently been proposed to cause lack of E-cadherin expression in the CRC cell line SW48 [27].

Finally, the present study confirms the association of *CTNNB1* mutations with LS associated CRC and further detected different hotspot loci according to the microsatellite status. All MSI-H CRCs displayed mutations affecting either codon 41 or 45 while mutations in MSS CRCs were more evenly distributed and included mutations within the β-TRcP binding site at residues 32–37 (Fig. 2). These findings correspond well to results of previous CRC sequencing studies reported by Hampel et al. and Yaeger et al. [28, 29]. In both studies, codons 41 and 45 of *CTNNB1* were found as mutational hotspots in MSI-H cancers while in MSS cancers larger deletions including the β-TRcP binding site dominated.

The reason for the selection of codon 41 or 45 mutations in MSI-H cancers is as unclear as the timing of *CTNNB1* first and second hit mutations in the colorectal adenoma to carcinoma sequence. If microsatellite instability precedes *CTNNB1* mutations as previously proposed by Ahadova et al. [30], the selection may be due to mutational events favoured by defective DNA-mismatch repair as already shown for mutations in *APC* [31]. On the other hand, several molecular studies on sporadic colorectal adenomas have revealed *CTNNB1* mutations preferentially at codons 41 and 45 at a significantly higher frequency than in CRC [31, 32]. Therefore it is possible that hetero- and homozygously *CTNNB1* mutated adenomas are at low risk to transform into cancer unless they acquire DNA mismatch repair deficiency as just the right mechanism to switch to malignancy.

## Conclusions

In conclusion, we herein show that *CTNNB1* mutations in CRC are frequently homozygous. Moreover, we found that the known association of *CTNNB1* mutations with LS-CRCs is restricted to mutations at codons 41 and 45, while in MSS CRCs mutations are more evenly distributed and commonly consist of larger in-frame deletions. This selection of specific mutations indicates a “just right” level of β-catenin stabilization to be essential for MSS- and MSI-H driven carcinogenesis. Consequently, aberrant activation of the Wnt signalling pathway might play different roles in the development and progression of MSS- and MSI-H-CRC. These findings are especially important in the context of evolving new target therapies of β-catenin which as yet, however, have not been incorporated into clinical practice [33, 34].

## Supplementary information

**Additional file 1: Supplementary Table S1.**

**Additional file 2.**

## Data Availability

The authors confirm that the data supporting the findings of this study are available within the article its supplementary materials. However, the datasets used and/or analysed during the current study are not publicly available due to data protection laws but are available from the corresponding author on reasonable request.

## Abbreviations

AF: Allele frequency
CRC: Colorectal cancer
CTNNB1: β-catenin
EMT: Epithelial-mesenchymal transition
IHC: Immunohistochemistry
LS: Lynch syndrome
MSI: Microsatellite instability/ microsatellite-unstable
MSS: Microsatellite stability/ microsatellite stable
