# Correction to: The majority of β-catenin mutations in colorectal cancer is homozygous

**DOI:** 10.1186/s12885-020-07645-z

**Published:** 2020-11-26

**Authors:** Alexander Arnold, Moritz Tronser, Christine Sers, Aysel Ahadova, Volker Endris, Soulafa Mamlouk, David Horst, Markus Möbs, Philip Bischoff, Matthias Kloor, Hendrik Bläker

**Affiliations:** 1 Institute of Pathology, Charité - Universitätsmedizin Berlin, Virchoweg 15 / Charitéplatz 1, 10117 Berlin, Germany; 2 Department of Applied Tumor Biology, Institute of Pathology, University of Heidelberg; Clinical Cooperation Unit Applied Tumor Biology, German Cancer Research Center (DKFZ); Molecular Medicine Partnership Unit (MMPU), University Hospital Heidelberg, Heidelberg, Germany; 3 Department of General Pathology, Institute of Pathology, University Hospital Heidelberg, Heidelberg, Germany; 4 Present address: Institute of Pathology, Universitätsklinikum Leipzig, Leipzig, Germany

**Correction to: BMC Cancer 20, 1038 (2020)**

**https://doi.org/10.1186/s12885-020-07537-2**

Following publication of the original article [1], the authors reported an error in the names of two of the authors.

*Incorrect names:*

Ayel Ahadova

Michael Kloor

*Correct names:*

Aysel Ahadova

Matthias Kloor

The original article [1] has been corrected.
