# Supplementary material for: The majority of β-catenin mutations in colorectal cancer is homozygous
Source: BMC Cancer. 2020 Oct 28;20:1038. doi: 10.1186/s12885-020-07537-2 (PMC7594410; doi:10.1186/s12885-020-07537-2)
Supplement: Supplementary file 1 — Additional file 1: Supplementary Table S1. [file 12885_2020_7537_MOESM1_ESM.docx]

Supplementary table 1

| Biallelic/Homozygous CTNNB1 mutations in CRCs | | | | | |
| --- | --- | --- | --- | --- | --- |
| Case | MSS/MSI-H | AA change | AF | Ref. mut | Relative AF |
| 3 | MSS | S45P | 68% | KRAS | 2.1 |
| 15 | MSS | T41A | 54% | PIK3CA | 2.1 |
| 1* | MSI-H | S45F | 50% | PIK3CA | 3.1 |
| 2 | MSI-H | S45F | 32% | PIK3CA | 2 |
| 8* | MSI-H | S45F | 62% | KRAS | 2.1 |
| 9* | MSI-H | S45P | 63% | FBXW7 | 2 |
| B-LS5 | MSI-H | T41A | 70% | FBXW7 | 1,9 |
| H-MS310 | MSI-H | T41A | 50% | KRAS | 2 |
| H-M783 | MSI-H | Del S45-G48 | 73% | KRAS | 2 |
| H-MSI 11 | MSI-H | S45F | 64% | PIKCA | 2.4 |
| H-MSI 26 | MSI-H | S45F | 60% | KRAS | 1.9 |
| B-LS3 | MSI-H | T41A | 40% | PIK3CA | 2 |
| H-M131 | MSI-H | T41A | 42% | KRAS | 1.9 |
| Biallelic/Homo- or hemizygous *CTNNB1* mutations in CRC | | | | | |
| 6 | MSS | del H24-H36 | 78% |  |  |
| 10 | MSS | delW25-I35 | 70% |  |  |
| 11 | MSS | S45F | 68% |  |  |
| 12 | MSS | Del A21-T41 | 62% |  |  |
| 5 | MSS | Del H24-H36 | 68% |  |  |
| 13* | MSI-H | T41A | 62% |  |  |
| H-MSI 8 | MSI-H | S45P | 72% |  |  |
| Monoallelic/ heterozygous *CTNNB1* mutations in CRCs | | | | | |
| 4 | MSS | G34R | 41% |  |  |
| 16 | MSS | S37C | 18% |  |  |
| 14 | MSI-H | S45F | 46% |  |  |
| 7 | MSI-H | delS45 | 48% |  |  |
| H-M691 | MSI-H | delS45 | 41% |  |  |
| H-MSI 28 | MSI-H | S45F | 47% |  |  |
| B-LS 6 | MSI-H | T41A | 44% |  |  |

Supplementary table 1. *CTNNB1* mutations and scoring for biallelic mutations. Case numbers without prefix correspond to CRCs from cohort 1, those beginning with either H or B to those from cohort 2.

Ref. mut.: gene with mutation used as reference

Relative AF: ratio of mutated allele frequency in *CTNNB1* and allele frequency of reference mutation

*loss of wild type *CTNNB1* confirmed by RNA sequencing
