# Supplementary figures and images for: The majority of β-catenin mutations in colorectal cancer is homozygous
Source: BMC Cancer. 2020 Oct 28;20:1038. doi: 10.1186/s12885-020-07537-2 (PMC7594410; doi:10.1186/s12885-020-07537-2)

## Slide 1
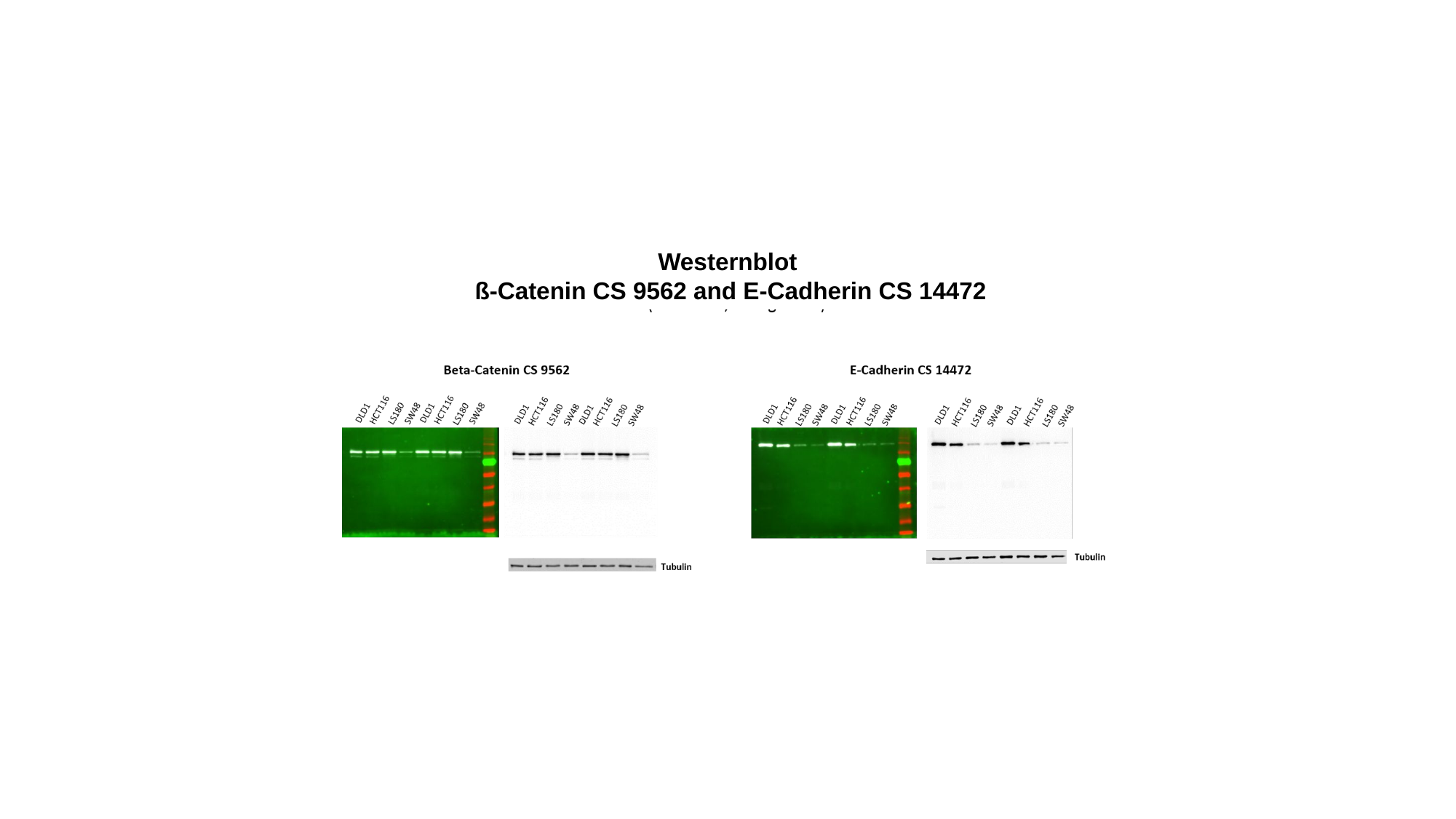

Westernblot
 ß-Catenin CS 9562 and E-Cadherin CS 14472

Supplement: Supplementary file 2 — Additional file 2. [file 12885_2020_7537_MOESM2_ESM.pptx]
